# Spatio-Temporal Heterogeneity of the Relationships Between PM_2.5_ and Its Determinants: A Case Study of Chinese Cities in Winter of 2020

**DOI:** 10.3389/fpubh.2022.810098

**Published:** 2022-04-11

**Authors:** Lu Yang, Song Hong, Chao He, Jiayi Huang, Zhixiang Ye, Bofeng Cai, Shuxia Yu, Yanwen Wang, Zhen Wang

**Affiliations:** ^1^School of Resource and Environment Science, Wuhan University, Wuhan, China; ^2^College of Resources and Environment, Yangtze University, Wuhan, China; ^3^Business School, The University of Sydney, Sydney, NSW, Australia; ^4^Center for Climate Change and Environmental Policy, Chinese Academy of Environmental Planning, Beijing, China; ^5^College of Resource and Environment, Huazhong Agricultural University, Wuhan, China; ^6^Economics and Management College, China University of Geosciences, Wuhan, China

**Keywords:** PM_2.5_, spatiotemporal heterogeneity, Chinese cities, COVID-19, GTWR

## Abstract

Fine particulate matter (PM_2.5_) poses threat to human health in China, particularly in winter. The pandemic of coronavirus disease 2019 (COVID-19) led to a series of strict control measures in Chinese cities, resulting in a short-term significant improvement in air quality. This is a perfect case to explore driving factors affecting the PM_2.5_ distributions in Chinese cities, thus helping form better policies for future PM_2.5_ mitigation. Based on panel data of 332 cities, we analyzed the function of natural and anthropogenic factors to PM_2.5_ pollution by applying the geographically and temporally weighted regression (GTWR) model. We found that the PM_2.5_ concentration of 84.3% of cities decreased after lockdown. Spatially, in the winter of 2020, cities with high PM_2.5_ concentrations were mainly distributed in Northeast China, the North China Plain and the Tarim Basin. Higher temperature, wind speed and relative humidity were easier to promote haze pollution in northwest of the country, where enhanced surface pressure decreased PM_2.5_ concentrations. Furthermore, the intensity of trip activities (ITAs) had a significant positive effect on PM_2.5_ pollution in Northwest and Central China. The number of daily pollutant operating vents of key polluting enterprises in the industrial sector (VOI) in northern cities was positively correlated with the PM_2.5_ concentration; inversely, the number of daily pollutant operating vents of key polluting enterprises in the power sector (VOP) imposed a negative effect on the PM_2.5_ concentration in these regions. This work provides some implications for regional air quality improvement policies of Chinese cities in wintertime.

## Introduction

At the end of 2019, with the sudden outbreak of COVID-19 in China, a series of containment measures were implemented by the government to limit the spread of infection. On 25 January 2020, the first day of the Chinese New Year, all cities in mainland China except cities in Tibet launched the highest level of emergency response (http://www.nhc.gov.cn/). These measures brought a temporary halt on human activities (1), e.g., transportation (2), industrial production (3) and energy consumption (4). The air quality during COVID-19 in China became much better than that in the same period of previous years (5). Particularly, the concentration of fine particulate matter (PM_2.5_) decreased by 10.5% (6). Thus, the stagnation caused by this epidemic provides a typical case study for exploring the important driving factors of haze pollution in Chinese cities in winter. The government can use this information to take more effective policy intervention in the future, specifically when addressing air quality improvement (7).

Numerous papers have studied the key factors influencing PM_2.5_ concentrations, including anthropogenic and natural factors (8). Anthropogenic factors include vehicle usage (9), industrial production (10), energy consumption (11), and household activities (12). In particular, the transportation sector is recognized as the largest contributor to the emissions of anthropogenic pollutants in urban air quality (13, 14), as this sector consumes a considerable amount of primary and secondary energy sources (15, 16). Previous studies have found that reasonable control measures policies for public transit improvement could effectively reduce PM_2.5_ concentrations (17). Industrial pollution has been found to be another major cause of the regional high PM_2.5_ concentrations in China (18). Massive fossil fuel combustion in industrial production is a notable source of PM_2.5_ emissions in Chinese cities (19). In terms of natural factors, meteorological conditions can influence the secondary formation of particulate matter by changing the atmospheric diffusion dilution conditions and material transformation process, thus affecting the regional haze pollution (20). Therefore, it is necessary to consider meteorological factors when studying the influencing factors of haze pollution (21, 22), particularly in a short time (23).

A large and growing number of research is devoted to analyzing air quality dynamics and its underlying driving factors after the COVID-19 outbreak (24). Many studies have mainly combined observations of air pollutant concentrations by remote sensing (1, 25) and numerical weather prediction models (5, 26) to reflect real-time changes in air quality. The contributions from various emission sources or changes in weather conditions have been common focuses of previous studies. For instance, Wang et al. (27) and He et al. (28) employed statistical models to evaluate the influence of different driving factors on air quality during the epidemic period, which emphasized the significant influence of restrictive measures. Yin et al. (29) and Shen et al. (30) both emphasized the exceptional importance of climate variability in regional air quality in China and noted that unfavorable meteorological conditions greatly contributed to the increase in the local PM_2.5_ concentration. However, the impacts of natural and anthropogenic factors on PM_2.5_ concentrations are dynamic and comprehensive (31, 32). Remote sensing data cannot directly represent human activities. As the lack of real-time socioeconomic data, the contribution of anthropogenic factors to pollutant changes is hard to quantify in such a short time. Although some papers have explored the contributions of travel restrictions to atmospheric environmental change during this epidemic with meteorological factors as control variables (7, 33), these studies have ignored the dynamic effects of natural factors and other human-induced emission sources on the PM_2.5_ concentration. In addition, because the PM_2.5_ concentration varies with space and time (6), an analysis of spatiotemporal heterogeneities on the impacts of different factors is needed.

Thus, in this paper, we collected higher temporal resolution data that could reflect human activities, that is, PM_2.5_ observations, natural factors data and anthropogenic factors data were matched at the same spatial and temporal scale. By using daily monitoring data of PM_2.5_ concentrations, mobility, and operating vents in prefecture-level cities across China during the COVID-19 epidemic, we could comprehensively analyse the real-time impact of human activities and natural conditions on haze pollution. In addition, the geographically and temporally weighted regression (GTWR) model was adopted to quantitatively evaluate the spatiotemporal heterogeneous in the relevance between driving factors and PM_2.5_ in the short term. Therefore, we could distinguish the main driving factors that dominate the temporal and spatial distribution of PM_2.5_ concentration in different cities in winter, the season with the most severe haze. That could provide a reference for the local government to put forward more effective haze control policies.

## Materials and Methods

### Study Periods

In the winter from 2019 to 2020, COVID-19 broke out in China. On January 23 of 2020, Wuhan, as the first city where COVID-19 cases appeared, announced that the city would lockdown to stop virus transmission. People in this city were not allowed to leave and advocated to stay at home. With the spread of the virus, other cities in China have gradually taken lockdown measures. Under this measure, human activities were greatly restricted, including the slowdown in the production of enterprises and the significant reduction in the travel of residents. This special period provides an unprecedented case for studying the impact of human activities on air pollutants.

### Methodology

#### The Univariate Local Moran's I Index

The univariate local Moran's I test was needed to assess spatial autocorrelation before we detected local agglomeration. The univariate local Moran's I index has been proven to be an effective tool for distinguishing the local spatial clusters, and it is calculated as shown in Equation (1):


(1) $$\begin{array}{l} \begin{array}{c} I_{i}=\frac{Y_{i}-\bar{Y}}{\sigma^{2}}\overset{n}{\underset{j=1,j\neq i}{\sum }}\left[\omega_{ij}\left(Y_{j}-\bar{Y}\right)\right]\; \end{array} \end{array}$$

Where *Y*_*i*_ is the value of the target variable (PM2.5 concentration) of the *i*-th city, *Y*_*j*_ represents the value of the PM2.5 concentration in other cities (*j*≠*i*), and $\bar{Y}$ denotes the average of the target variable. σ^2^ is the variance of the target variable. ω_*ij*_ is the spatial weight displaying the neighboring relations among the geographical units. In this research, we used GeoDa software to obtain the LISA for cities' PM2.5 concentrations before and after the COVID-19 outbreak.

#### The GTWR Model

Differentiated from the traditional time series model and spatial econometric model, the GTWR model can comprehensively consider the temporal correlation and the spatial characteristics (34), better capturing the spatial and temporal heterogeneity of statistical correlations among regression variables. The PM_2.5_ concentration may accompany spatial heterogeneity (35) and time-series relevance, particularly in the short term (36). We used this model to analyse the spatiotemporal heterogeneity of impacts of natural and anthropogenic factors on PM_2.5_ pollution.


(2) $$\begin{array}{l} \begin{array}{c} \ln\;Y_{i}=\beta_{0}\left(u_{i},v_{i},t_{i}\right)+\underset{k}{\sum }\beta_{k}\left(u_{i},v_{i},t_{i}\right)\ln\;X_{ik}+\varepsilon_{i} \end{array}\; \end{array}$$

Where *Y*_*i*_ is the PM2.5 concentration of the *i*-th city, and *X*_*ik*_ is the influencing factor of the *i*-th city on the *k*-th day, including natural and anthropogenic factors. (*u*_*i*_, *v*_*i*_, *t*_*i*_) denotes the *i*-th city's geographical position (*u*_*i*_, *v*_*i*_) and time location (*t*_*i*_). β_*k*_(*u*_*i*_, *v*_*i*_, *t*_*i*_) represent the coefficients of space-time observation sample *i*, which can be explained as the percentage of dependent variable (*Y*) change with 1% variation of the independent variable (*X*). ε_*i*_ is the random error term.

β_*k*_(*u*_*i*_, *v*_*i*_, *t*_*i*_) for variable *k* and spatiotemporal location *i* is examined by the following equation:


(3) $$\begin{array}{l} \begin{array}{c} \hat{\beta }\left(u_{i},v_{i},t_{i}\right)={\left[X^{T}W\left(u_{i},v_{i},t_{i}\right)X\right]}^{-1}X^{T}W\left(u_{i},v_{i},t_{i}\right)Y\; \end{array}\; \end{array}$$

Where *W*(*u*_*i*_, *v*_*i*_, *t*_*i*_) is the spatiotemporal weight matrix estimated by the temporal and spatial distances and the decay functions. Therefore, the spatiotemporal weight for independent variable needs to be calculated before conducting this model analysis. The spatiotemporal distance *d*^*ST*^ could be split into spatial distance (*d*^*S*^) and temporal distance (*d*^*T*^), as shown in Equation (4):


(4) $$\begin{array}{l} \begin{array}{c} d^{ST}=\text{λ}d^{S}+\mu d^{T}\; \end{array}\; \end{array}$$

Where λ and μ are scale factors to measure the impacts of the spatial and temporal distance in their respective metric systems. Then, the spatiotemporal distance *d*_*ij*_ between cities *i* and *j* is calculated by Equation (5):


(5) $$\begin{array}{l} \begin{array}{c} d_{ij}^{ST}=\sqrt{\text{λ}\left[{\left(u_{i}-u_{j}\right)}^{2}+{\left(\upsilon_{i}-\upsilon_{j}\right)}^{2}\right]+\mu {\left(t_{i}-t_{j}\right)}^{2}}\; \end{array}\; \end{array}$$

Thus, the spatiotemporal weight (α_*ij*_) can be obtained by using Equation (6):


(6) $$\begin{array}{l} \begin{array}{c} \alpha_{ij}=\exp\left(-\frac{{\left(d_{ij}^{ST}\right)}^{2}}{b_{ST}^{2}}\right)\; \end{array}\; \end{array}$$

Where *b*_*ST*_ represents the non-negative parameter of the spatiotemporal bandwidth. In this work, cross-validation via the minimum sum of the squared error [*CVRSS*(*h*)] was used to find the optimal spatiotemporal bandwidth (37).


(7) $$\begin{array}{l} \begin{array}{c} CVRSS\left(b\right)=\underset{i}{\sum }{\left(y_{i}-{ŷ}_{\neq 1}\left(b\right)\right)}^{2}\; \end{array}\; \end{array}$$

Where ŷ_*i*_(*b*) denotes the predicted value from the GTWR model, and the function is the sum of squared errors.

#### Stability Estimation of Coefficients

We applied the Kernel function to assess the stability of the correlation coefficients (18). The density function of the variable *x* is shown in Equation (8):


(8) $$\begin{array}{l} \begin{array}{c} f\left(x\right)=\frac{1}{Nh}\overset{n}{\underset{i=1}{\sum }}K\left(\frac{X_{i}-\bar{X}}{h}\right)\; \end{array}\; \end{array}$$

Where *X*_*i*_ is the coefficients subordinated to independent and identical distributions. *n* denotes the number of *X*. *h* is the bandwidth, and $\bar{X}$ is mean value. The Epanechnikov function widely used by previous studies was adopted as the kernel function for estimation in this work.

### Data Source

#### PM_2.5_ Concentrations

Hourly observations of the PM2.5 concentrations at 1,672 monitoring sites in our study period were collected from the real-time monitoring data system of the China National Environmental Monitoring Center (CNEMC) (https://air.cnemc.cn:18007/). To ensure the continuity and reliability of the original data, we conducted strict data quality control on the hourly PM_2.5_ concentration data before analysis according to the provisions on the validity of air pollutant concentration data (GB 3095-2012) (https://www.mee.gov.cn/), and we removed abnormal data. The daily average of each site and then averaged these daily averages of sites in each city was calculated as the daily PM_2.5_ concentration of the city. Based on the data pre-processing, 332 cities were selected in this study to discuss regional changes in PM_2.5_ concentrations.

#### Natural Factors

Four essential meteorological factors were selected to report the natural impacts on PM_2.5_, including surface pressure (sp, KPa), temperature (tem, K), relative humidity (rh, %), and wind speed (ws, m/s). In this work, rh was calculated by the dewpoint temperature (d_2m_) and temperature (t_2m_) based on the Clapeyron-Clausius equation. ws is the square root of the sum of the squares of the eastward component of the 10 m wind (u_10_) and the northward component of the 10 m wind (v_10_). The reanalysis dataset (ERA5-Land hourly data) for these data was obtained from the European Centre for Medium-Range Weather Forecasts (ECWMF) (https://www.ecmwf.int/). This reanalysis dataset is hourly 0.1 × 0.1 grid data. The city-scale meteorological data processing method is similar to that used for the PM_2.5_ concentrations.

#### Anthropogenic Factors

In this study, we used the intensity of trip activities as an indicator to assess traffic flow at each site during the COVID-19 epidemic (7, 33, 38). This intensity of trip activities (ITAs), an exponential result of the ratio calculated by the number of people who travel in the city divided by the resident population in the city, can be used to measure the daily passenger traffic pressure on the urban transport system (https://qianxi.baidu.com/). Therefore, we applied this index to indirectly reflect the impact of human travel and lifestyle changes on PM_2.5_ concentration (39, 40).

Changes in industrial production affected by COVID-19 were characterized by the numbers of daily pollutant operating vents of key polluting enterprises in the industrial sector (VOI), which could reflect the dynamic change in industrial pollution intensity. The numbers of daily pollutant operating vents of key polluting enterprises in the power sector (VOP) were used to report the variation in energy consumption. The pollutant operating vents were the sum of the vent numbers of dust, nitrogen oxide (NOx) and sulphur dioxide (SO_2_). After the Spring Festival in 2020, under the strict implementation of epidemic control measures, the Chinese government announced that some essential enterprises could resume production with restrictions. We thus collected the daily pollutant operating vent numbers of more than 9,000 key polluting enterprises in China from January to February 2020 (http://www.ipe.org.cn/). These enterprises usually pose great environmental risks, such as a great quantity these air pollutants, and they are therefore screened by the environmental protection department of the local government based on the environmental quality improvement requirements of their respective administrative areas and other specified conditions (41). The key polluting enterprises in the power sector was screened according to the national economic industry classification (GB/T4754-2017). We searched each enterprise name and check the industry classification of the enterprise one by one. Moreover, combined with Google Maps, we have verified the spatial coordinates of enterprises point by point to ensure the accuracy of the number of enterprise vents in each city.

The descriptive statistics for each variable are presented in Table 1.

**Table 1:** The descriptive statistics for each variable.

**Variable**	**Number**	**Mean**	**Std. Dev**.	**Max**	**Min**
PM_2.5_ (μg/m^3^)	13,944	55.97	45.94	549.02	2.33
ITAs	13,944	3.79	1.58	8.88	0.36
VOI	13,944	59.0	166.3	2,100	0
VOP	13,944	26.5	56.3	372	0
sp (KPa)	13,944	93.94	10.06	103.93	53.74
temp (K)	13,944	275.84	10.03	299.46	248.41
rh (%)	13,944	69.25	15.80	98.54	10.70
ws (m/s)	13,944	1.65	1.11	10.51	0.01

## Results

### Spatiotemporal Changes in PM_2.5_ Concentrations

**Figure 1:**
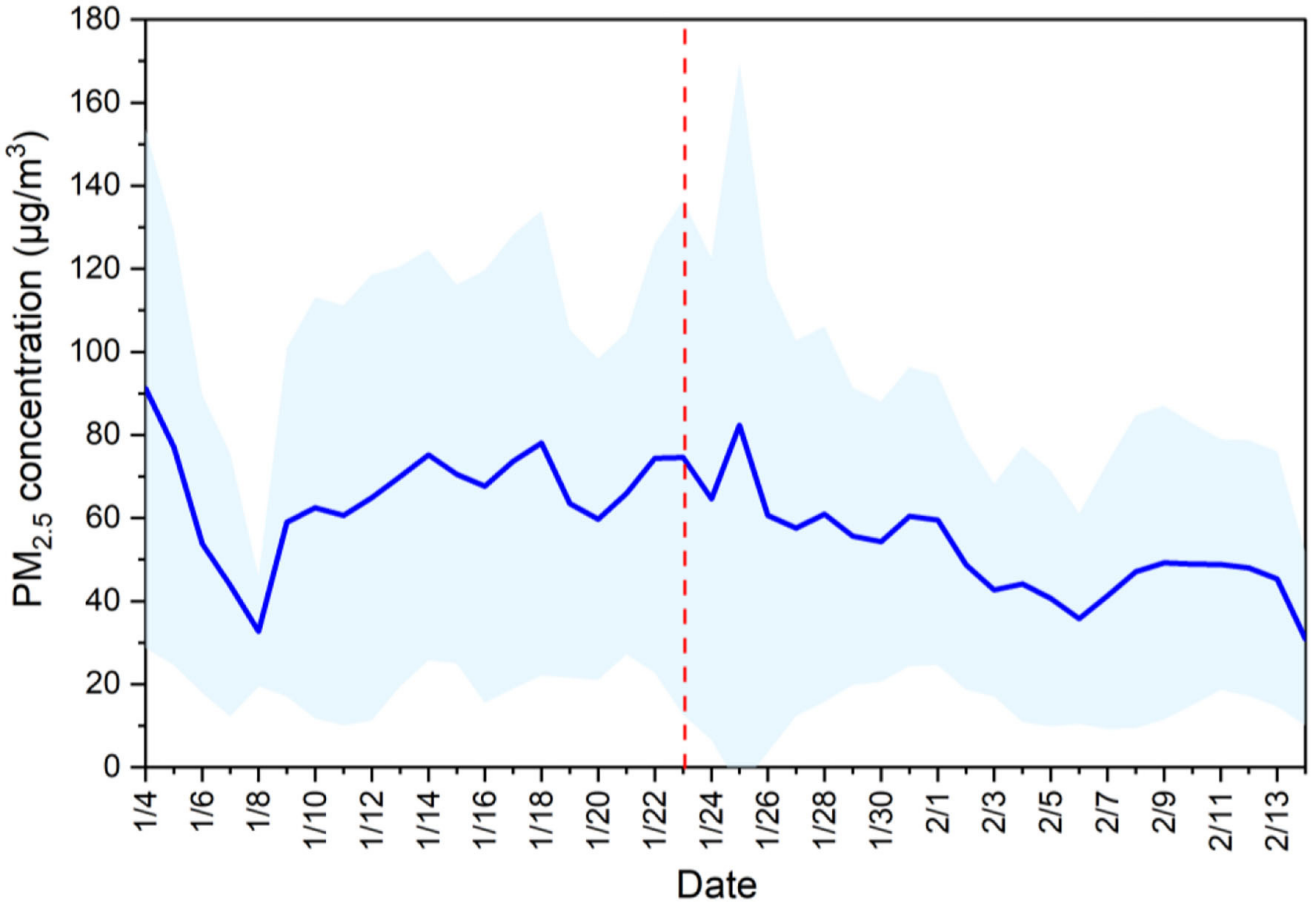
Time-variation in daily PM_2.5_ concentrations Chinese cities before and after lockdown measures were implemented in response to the outbreak of COVID-19. The red line represents the day that lockdown measures began in Wuhan, and shaded blue denote the standard deviations.

The time-variation of daily PM_2.5_ concentrations in Chinese cities before and after lockdown measures were implemented are shown in Figure 1. Before the measures were taken, the daily average PM_2.5_ concentration reaching its minimum value (32.7 μg/m^3^) on Jan 8, rapidly returned to 60 μg/m^3^, and continued to fluctuate upward. In contrast, as cities in China gradually locked down, the daily average national PM_2.5_ concentrations showed a clear trend of first increasing and then decreasing, with the value declining to 30.9 μg/m^3^ on February 14. This tendency was consistent with the findings of Wang et al. (42).

**Figure 2:**
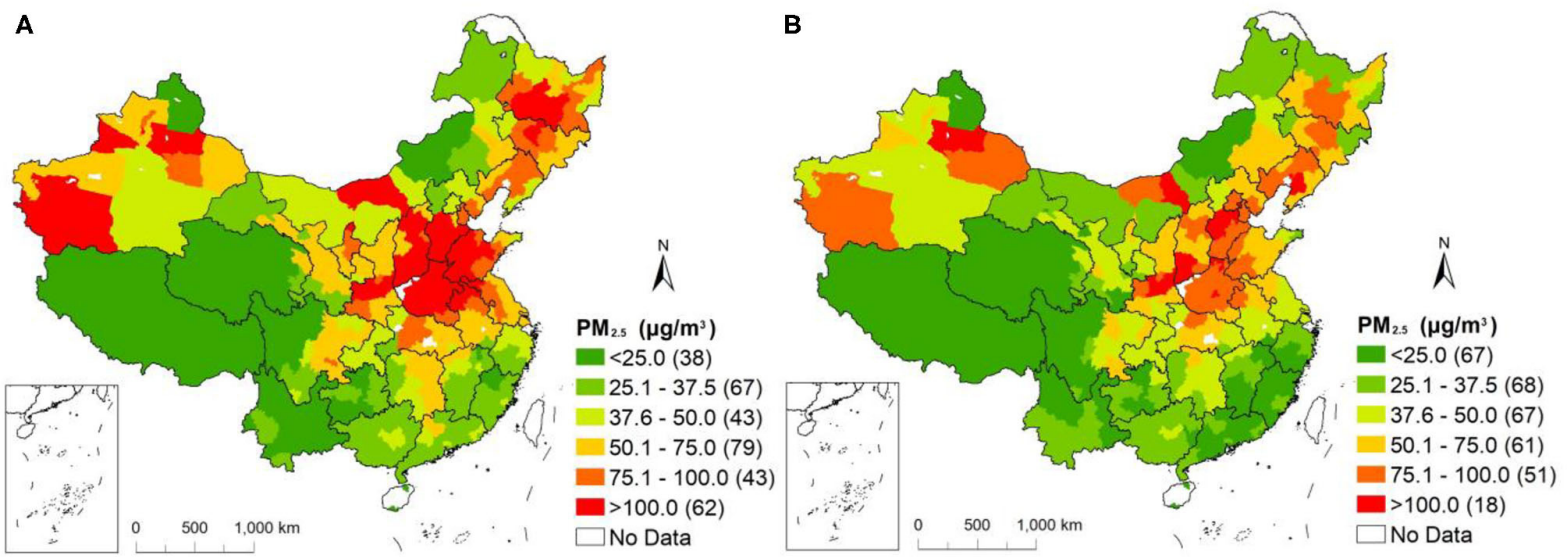
Distributions of daily PM_2.5_ concentrations in Chinese cities before **(A)** and after **(B)** lockdown measures taken in response to the outbreak of COVID-19.

The daily average PM_2.5_ concentrations for cities before and after lockdown measures were taken presented diverse spatial distribution characteristics (Figure 2). In the absence of any measures to limit human activities, the 105 cities with higher PM_2.5_ concentrations, more than 75 μg/m^3^, were concentrated in Northeast China (Heilongjiang, Jilin and Liaoning Provinces), the North China Plain (Tianjin, Hebei, Shanxi, Henan, Shandong Provinces and northern Anhui Province), and the Tarim Basin (Xinjiang Province). Other areas with high PM_2.5_ concentrations (50–75μg/m^3^) were distributed around the regions with the highest concentrations. However, after cities gradually locked down, 84.3% of cities experienced a decline in PM_2.5_ concentration. The number of cities characterized by PM_2.5_ concentrations higher than 75 μg/m^3^ was significantly reduced, decreasing by 34.2%. Similar to the period before measures were taken, cities with high PM_2.5_ concentrations were concentrated in Northeast China, the North China Plain and Xinjiang Province. Moreover, haze pollution was improved in 30 cities, with PM_2.5_ concentrations <37.5 μg/m^3^. These cities were mainly distributed in the southern coastal areas.

**Figure 3:**
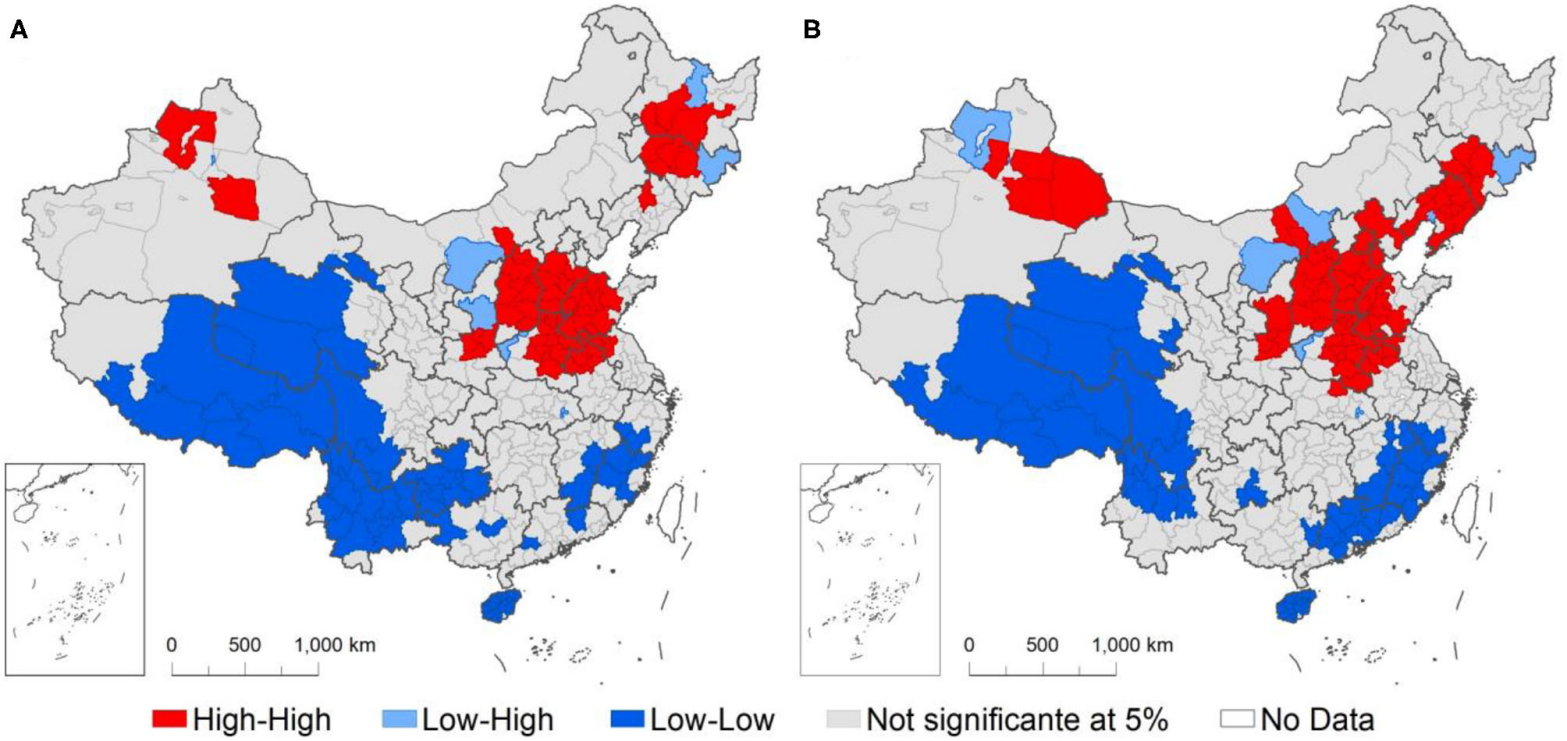
The univariate Local Moran's I for PM_2.5_ concentrations in Chinese cities before **(A)** and after **(B)** lockdown measures were taken in response to the outbreak of COVID-19.

We further conducted a local Moran's I test (Figure 3) to identify the spatial clustering characteristics of the PM_2.5_ concentration. During our study period, cities' daily PM_2.5_ concentrations presented an appearance of regional convergence, mainly marked by a high-high group and a low-low group. When human activities were not limited, the high-high group occurred in Northeast China, the North China Plain, and the Tarim Basin, which indicated that the PM_2.5_ pollution in these regions was more serious and that the positive local spatial autocorrelation of PM_2.5_ existed in these cities. Cities with low PM_2.5_ concentrations were mainly distributed in Xizang, Qinghai, Sichuan, Yunnan, Guizhou, and Fujian Provinces, suggesting that the haze pollution in these areas was relatively light. The local spatial autocorrelation of haze pollution also existed in these places but the correlation was weak. However, after lockdown measures were taken, the club-convergence phenomenon became more obvious, with the high-high cluster in Northeast China shifting to the south. The regions characterized by the low-low cluster in Northwest China shrank, while the regions in Southwest China expanded to Guangdong Province.

### Impacts of Factors on PM_2.5_ Concentrations

The GTWR model was employed to calculated the influence of selected factors on cities' haze pollution in this study, and the parameters of the model are displayed in Table 2. In the simulation results of the model, the *R*^2^ value was 0.403, the adjusted *R*^2^ was 0.402, the bandwidth value was 0.115, the sigma value was 0.259, the CV value was 808.202 and the spatiotemporal distance ratio was 1.000.

**Table 2:** Regression parameters of the GTWR model.

**Regression parameter**	**Value**
Bandwidth	0.115
Residual squares	934.863
Sigma	0.259
CV	808.202
*R^2^*	0.403
Adjusted *R^2^*	0.402
Spatiotemporal distance ratio	1.000

#### Stability of Coefficients

**Figure 4:**
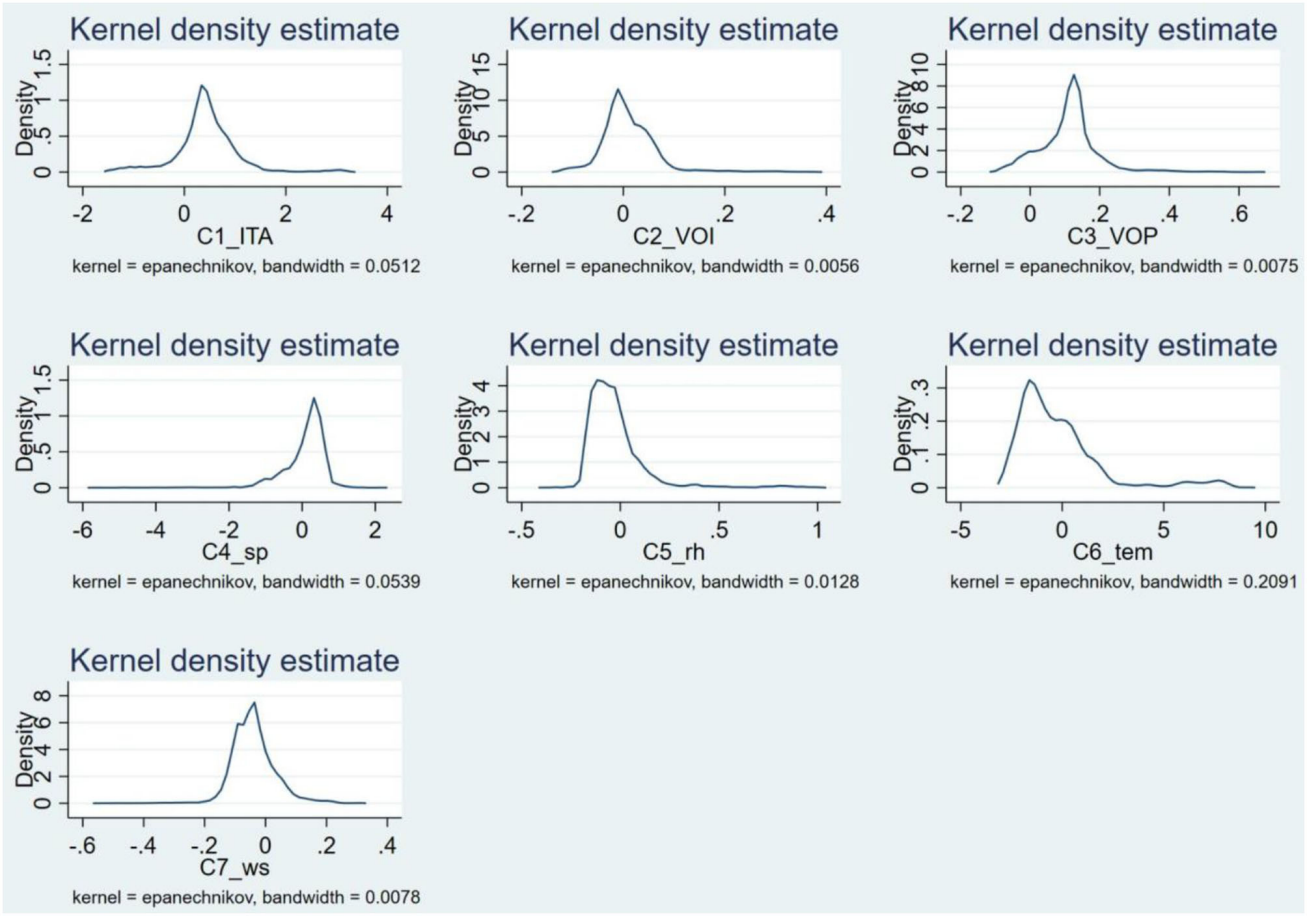
Kernel density distribution of each variable coefficient.

From the Kernel distribution of coefficients of different variables (Figure 4), we can see that the coefficients of ITAs, VOI and VOP were concentrated at approximately 0.50,−0.01, and 1.40, respectively. This result indicates that in most cities, the increase in ITAs and VOP had a promotion effect on haze pollution in the winter of 2020, while the increase in VOI had the opposite effect. Among the four natural factors we analyzed, the largest density of coefficients of sp was distributed at 0.25, which illustrates that with the increase in sp, PM_2.5_ concentrations in most cities were promoted. In contrast, the coefficients of rh, tem and ws were left-distributed, and the peaks emerged at approximately−0.08,−1.25, and−0.25, respectively, revealing that the increase in rh, tem and wind would restrain the raise of PM_2.5_ concentration in most cities during our study period.

#### Impacts of Natural Factors

**Figure 5:**
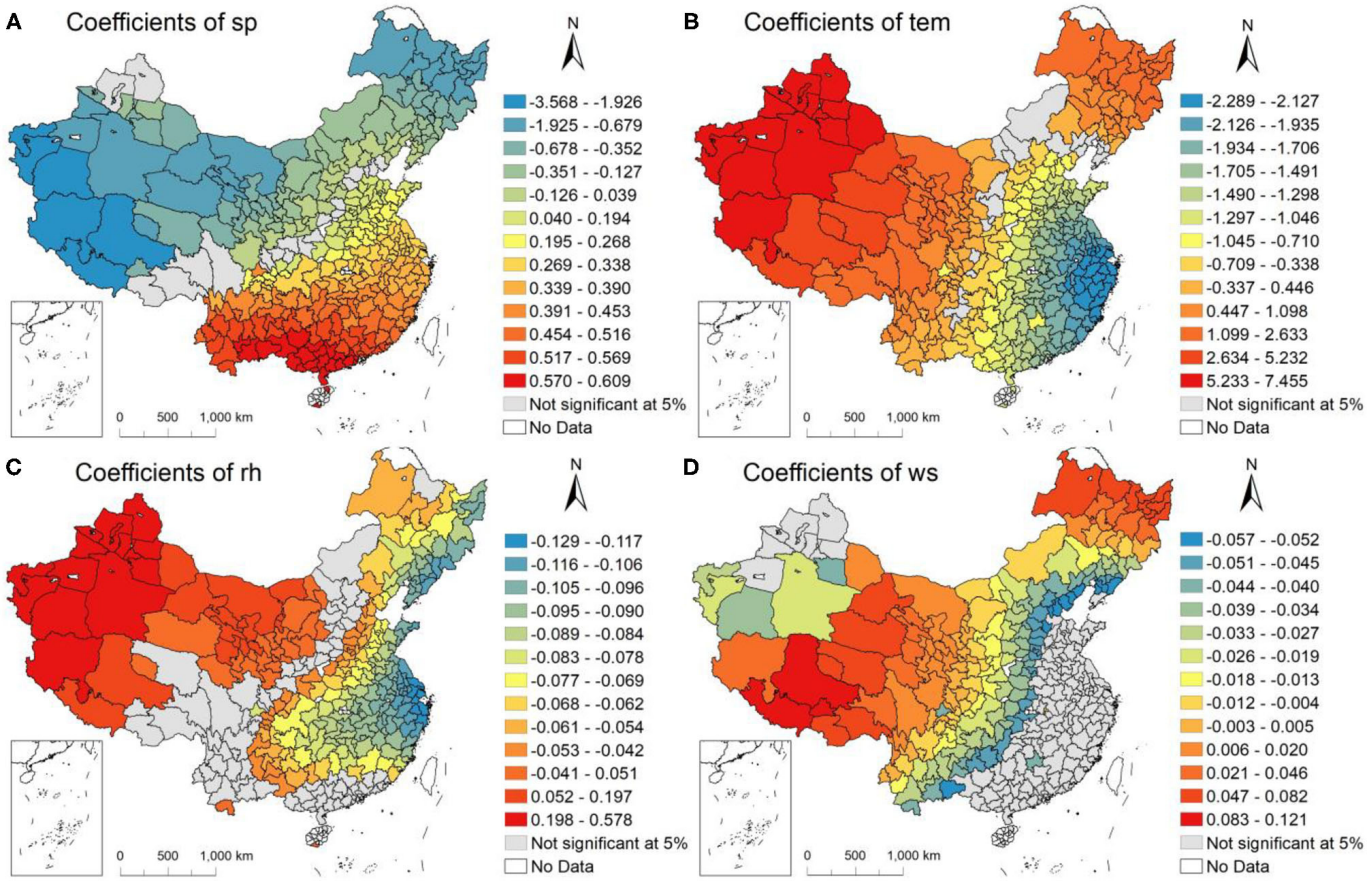
Results of natural factors from the GTWR in 332 Chinese cities. **(A)** Coefficients of surface pressure (sp), **(B)** coefficients of temperature (tem), **(C)** coefficients of relative humidity (rh), and **(D)** coefficients of wind speed (ws).

The surface pressure (sp) coefficients for the 332 Chinese cites during our study period (Figure 5A) showed an increasing trend of coefficients of the sp, with city moving from the north to the southwest of the country. Cities in South China experienced a positive relationship between the sp and PM_2.5_ concentrations, that the increased sp in the south aggravated the PM_2.5_ concentrations, with Fangchengganng (0.609), Haikou (0.608) and Chongzuo (0.608) ranking among the top three cities. This is mainly because increased sp hindered the diffusion of pollutants in the vertical direction (43). However, an increased sp would limit PM_2.5_ pollution in northern cities, and the lowest coefficients appeared in the Ali (-3.568). Decreases of PM_2.5_ concentration in these cities was resulted from specific meteorological conditions formed by sp and other meteorological factors (44).

The coefficient of temperature (Figure 5B) shown a negative association with the PM_2.5_ concentrations in southeast and middle regions of the country, which revealed that the increased temperature in these areas would bring with a reduction in PM_2.5_ levels. Among the cities, Wenzhou was found to preserve the most significant negative correlation between temperature and PM_2.5_ concentration, a related coefficient being−2.289. As higher surface temperature would intensify atmospheric convection, PM_2.5_ concentration in these cities reduced. In contrast, positive correlations between temperature and PM_2.5_ concentration was exhibited in north regions. The temperature in the Ali exerted the most positive impact on the PM_2.5_ concentration, with the correlation coefficient being 7.455. The winter temperature in northern cities was low (Supplementary Figure 1), although it increased during the study period, resulting in an inversion layer in the atmosphere, which made PM_2.5_ difficult to diffuse (32).

From the spatial distribution of relative humidity (rh) coefficients (Figure 5C), we can see those regions with the most remarkable linkage between rh and PM_2.5_ were mostly situated in northwest regions, including Yili Kazak (0.578), Aksu (0.577), and Bortala Mongolia (0.572), among others. In the environment with high rh, water vapor in the air was easy to condense water-droplets, which would lead to the growth of moisture absorption of particles and aggravate haze pollution (45). Zhoushan (- 0.129), Karamay (- 0.124) and other cities in eastern areas displayed negative associations between rh and haze pollution. Whereas, the absolute value of these negative correlation coefficient was small, indicating that the reduction of rh in these areas had limited effect on the increase of PM_2.5_ concentration in this study period.

The spatial distribution of wind speed (ws) coefficients (Figure 5D) showed that the relations between ws and PM_2.5_ concentrations were not significant in the eastern and southern regions during our study period. Among the cities with a significant relation, the ws coefficients of several cities located in West and Northeast China were positive, but the coefficients were found to be negative in other cities. Specifically, the wind speed in Shigatse had the greatest promotion of haze pollution, with a coefficient of 0.121. The lowest ws coefficient emerged in Yingkou (- 0.057). As one of the important functions of wind was to transport air pollutants, a high wind speed contributed to the air pollutants' dilution and diffusion process, resulting in decreased regional PM_2.5_ concentrations (46).

#### Impacts of Anthropogenic Factors

**Figure 6:**
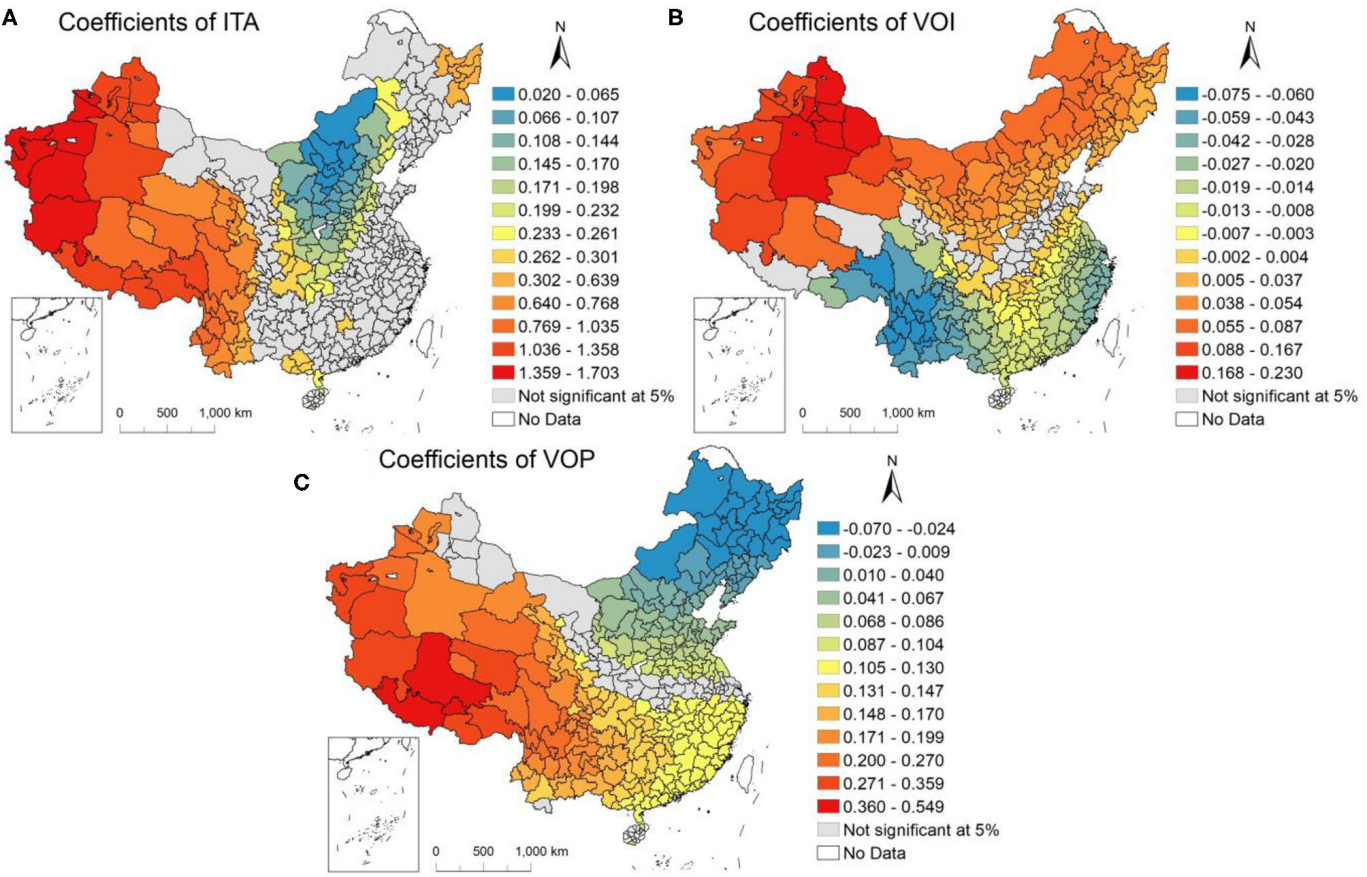
Results of anthropogenic factors from the GTWR in 332 Chinese cities. **(A)** Coefficients of the intensity of trip activities (ITA), **(B)** coefficients of the numbers of daily pollutant operating vents of key polluting enterprises in the industrial sector (VOI), and **(C)** coefficients of the numbers of daily pollutant operating vents of key polluting enterprises in the power sector (VOP).

Distribution of the correlation coefficient of three anthropogenic factors considered in this work is shown in Figure 6. The correlation of the ITAs and PM_2.5_ concentrations (Figure 6A) was not significant in approximately half of the cities located in the eastern regions during our study period. This result was in accordance with previous studies conducted by Zeng and Bao (47) and Bao and Zhang (33). In addition, the correlation parameters for ITAs were found to be significantly positive in other cities, and the coefficients in the western and north-eastern regions were higher than those in the northern and central areas. The ITAs in Kashgar was found to enforce the greatest promotion impact on the PM_2.5_, and every 1% raise in ITAs would cause a 1.703% decrease in PM_2.5_ concentration, followed by Kizilsu Kirgiz, Ali and Hotan.

As seen from Figure 6B, the correlation between the VOI and PM_2.5_ concentrations was gradually increase as area shifted from north-eastern and north-western parts to eastern and southwestern region (excepted for some cities in Shandong, Henan, Hubei, Shanxi, Ningxia, Qinghai and Tibet Provinces). Correlation coefficients in the southwestern and south-eastern coastal cities were less than zero, suggesting that raise of the VOI were accompanied by the decline of PM_2.5_ concentrations. Cities in northern China, the PM_2.5_ concentration was shown increase with grow in VOI. The VOI in Diqing Tibetan played a particularly substantial role in decreasing the PM_2.5_ concentration, with the coefficient of−0.075, then, the Nujiang Lisu Nationality Autonomous region (- 0.074), Lijiang (- 0.074), and Dali (- 0.071). The VOI in Turpan exhibited the greatest promoting influence on the PM_2.5_ concentration, having the coefficient of 0.230.

Contrary to the VOI results, the coefficient of VOP demonstrated an upward trend as cities shifted from the northeast to the southwest of China (Figure 6C). In north-eastern cities, the VOP was shown to more obviously restrain PM_2.5_ concentrations than in other cities. Hulun Buir (-0.070) had the lowest coefficient, followed by Heihe (-0.056) and Qiqihar (-0.053). The significance of the positive influence of VOP on PM_2.5_ concentrations in Shigatse, Lhasa, and Nagqu ranked as the top three among cities, with coefficients of 0.549, 0.443, and 0.419, respectively.

## Discussion

During the study period, the areas with serious haze pollution in China were mainly concentrated in the North China Plain (NCP), the north of the Yangtze River Delta (YRD), Northeast China (NEC) and some cities in Xinjiang. The specific area is shown in Supplementary Figure 1, and relative change rates of 7 driving factors in these areas before and after city closure are displayed in Supplementary Table 1. According to the analysis results, the spatial heterogeneity of the relationship between natural factors and PM_2.5_ concentration was obvious. The decrease of PM_2.5_ concentration in NCP was mainly caused by the increase of temperature and wind speed under the meteorological conditions at that time. Compared with other natural factors, the rise of temperature was the main reason for the mitigation of haze pollution in the YRD. In most cities in NEC, the reduction of relative humidity was the most important natural factor to reduce haze pollution. In addition, the main natural factor causing the decrease of PM_2.5_ concentration in some cities in Xinjiang was the increase of wind speed.

We also analyzed the spatiotemporal heterogeneity of the impacts of human activities on the PM_2.5_ concentration during our study period. The intensity of trip activities (ITAs) presented a positive effect on haze pollution, denoting that the limitation of travel intensity would generally reduce the PM_2.5_ concentration (33, 42). The coefficient distribution suggested that the aggravating effects of ITAs on haze pollution in western areas were more significant than those in central regions. Regions with significantly positive correlation coefficients in VOI were distributed in northern China, in which the PM_2.5_ concentrations were generally high. By contrast, the of VOP with PM_2.5_ concentrations showed an uptrend, as areas shifted from the northeast to the southwest. It is worth noting that the low VOP coefficients were concentrated in areas with winter heating (48). This result means that in China, some achievements have been made in using clean energy heating to improve air quality in winter (49).

In our study, we selected the period of COVID-19 outbreak in China as a case and calculated the relevance between city-level PM_2.5_ concentration and its driving forces during that short time, exploring variation characteristics of these correlations in time and space dimensions. According to the results, we found that it is possible to propose regional targeted air quality improvement suggestions for winter by considering spatiotemporal heterogeneity in the interactivities of PM_2.5_ concentrations and human activities. Although the travel restrictions implemented after the COVID-19 outbreak cannot be applied to air pollution management and control, it is feasible to improve haze pollution by declining unnecessary individual movements and increasing people's awareness of green commuting (50). When planning to improve air quality in northern areas in winter (with positive VOI correlation coefficients), the operating vent number of enterprises in the industrial sector with emission levels more than the regional standard limits should be strictly restrained or even eliminated. However, cities located in the west and south of the country, where the correlation of the VOP with PM_2.5_ concentrations is positive, must pay more attention to the reduction in pollutant emissions of polluting enterprises in the power sector (51). In addition, although the recession has caused a temporary decrease in air pollution, it would be difficult to preserve this decline after national labor force gradually went back to work. Therefore, in the future, green production and consumption will be a good way to improve air pollution without reducing people's normal production and living needs (52, 53).

This study has limitations. The available daily dynamic data of anthropogenic factors influencing PM_2.5_ distribution during this epidemic period were limited. The indicators we selected did not cover the influences of all anthropogenic factors. Because of absence of pollutants emissions data, some uncertainty existed in the process of evaluating the contributions of different sectors to the PM_2.5_ distributions by using indirect indicators. However, we used these indicators to reflect the dynamic changes in human activities, and the contribution of pollutant emissions to the PM_2.5_ concentration distribution was not discussed in this paper.

## Conclusions

After the strict implementation of epidemic control measures gradually taken in Chinese cities, 84.3% of cities' PM_2.5_ concentrations decreased. Spatially, in the winter of 2020, cities with higher PM_2.5_ concentrations (>75 μg/m^3^) were mostly situated in Northeast China, the North China Plain and the Tarim Basin. As the problem of haze pollution was suppressed, the spatial aggregation of PM_2.5_ concentration was more prominent. The surface pressure coefficients for Chinese cities increased from the north to the southwest of China. The coefficient of temperature exhibited negative correlations with PM_2.5_ concentrations in southeast and middle cities; inversely, positive relationships were found in the northeast and northwest regions. Cities with higher relevance of PM_2.5_ concentrations with relative humidity were mainly situated in Northwest China. Regions in western and north-eastern China witnessed a significant positive influence of wind speed on PM_2.5_ concentrations. Among the three anthropogenic factors we considered, the ITAs exhibited significant positive influence on haze pollution, and the correlation maintained a high intensity during the study period. The VOI coefficients in northern cities were positive, while those in south-western and south-eastern coastal cities shown significantly negative. In addition, the VOP coefficients demonstrated an uptrend as region shifted from the northeast to the southwest of the country. Based on the spatiotemporal heterogeneity of coefficients for each factor, policy for controlling haze pollution need to be pointed out by considering the distinct meteorological and human activities at regional scales. Reasonable restrictions on people's travel intensity can effectively prevent haze pollution. In winter, cities in the north could focus on eliminating the operating vents of enterprises in the industrial sector with emission levels exceeding the regional standard limits, while cities in the west and south could pay more attention to the reduction of unnecessary pollutant emissions of polluting enterprises in the power sector.

## Data Availability Statement

Publicly available datasets were analyzed in this study. Data of intensity of trip activities can be found here: https://qianxi.baidu.com/. The numbers of daily pollutant operating vents of key polluting enterprises can be found here: http://www.ipe.org.cn/. Meteorological data can be found here: https://www.ecmwf.int/, and observations of PM_2.5_ concentrations can be found here: https://air.cnemc.cn:18007/.

## Author Contributions

LY: formal analysis, data curation, writing-original draft, and writing-review and editing. SH, SY and ZW: writing-review and editing. CH: writing-review and editing and resources. JH: data curation. ZY: data curation and validation. BC: resources. YW: writing-original draft. All authors contributed to the article and approved the submitted version.

## Conflict of Interest

The authors declare that the research was conducted in the absence of any commercial or financial relationships that could be construed as a potential conflict of interest.

## Publisher's Note

All claims expressed in this article are solely those of the authors and do not necessarily represent those of their affiliated organizations, or those of the publisher, the editors and the reviewers. Any product that may be evaluated in this article, or claim that may be made by its manufacturer, is not guaranteed or endorsed by the publisher.
