# Supplementary figures and images for: Spatio-Temporal Heterogeneity of the Relationships Between PM2.5 and Its Determinants: A Case Study of Chinese Cities in Winter of 2020
Source: Front Public Health. 2022 Apr 11;10:810098. doi: 10.3389/fpubh.2022.810098 (PMC9035510; doi:10.3389/fpubh.2022.810098)

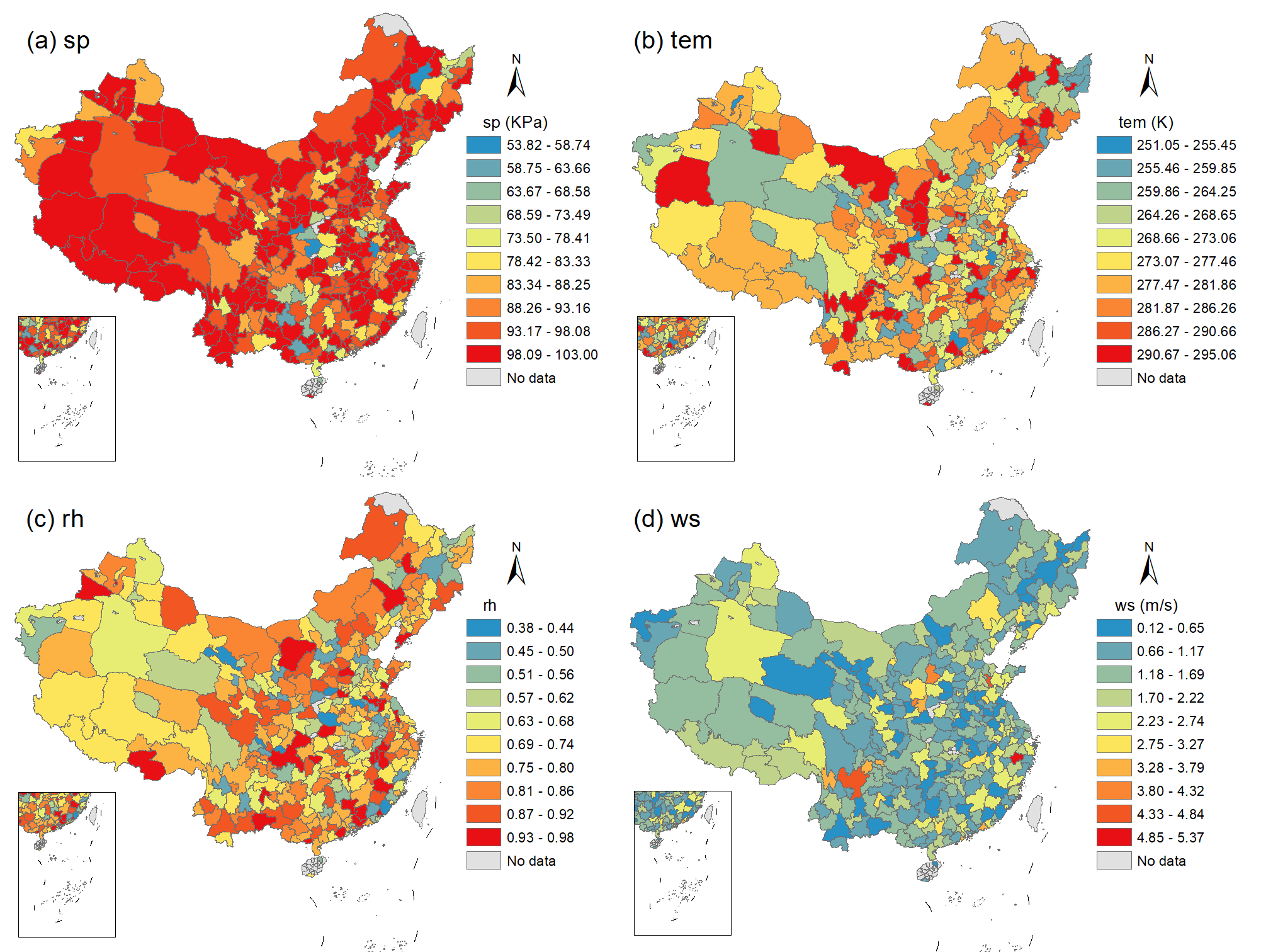

Supplement: Supplementary file 1 [file Image_1.TIF]

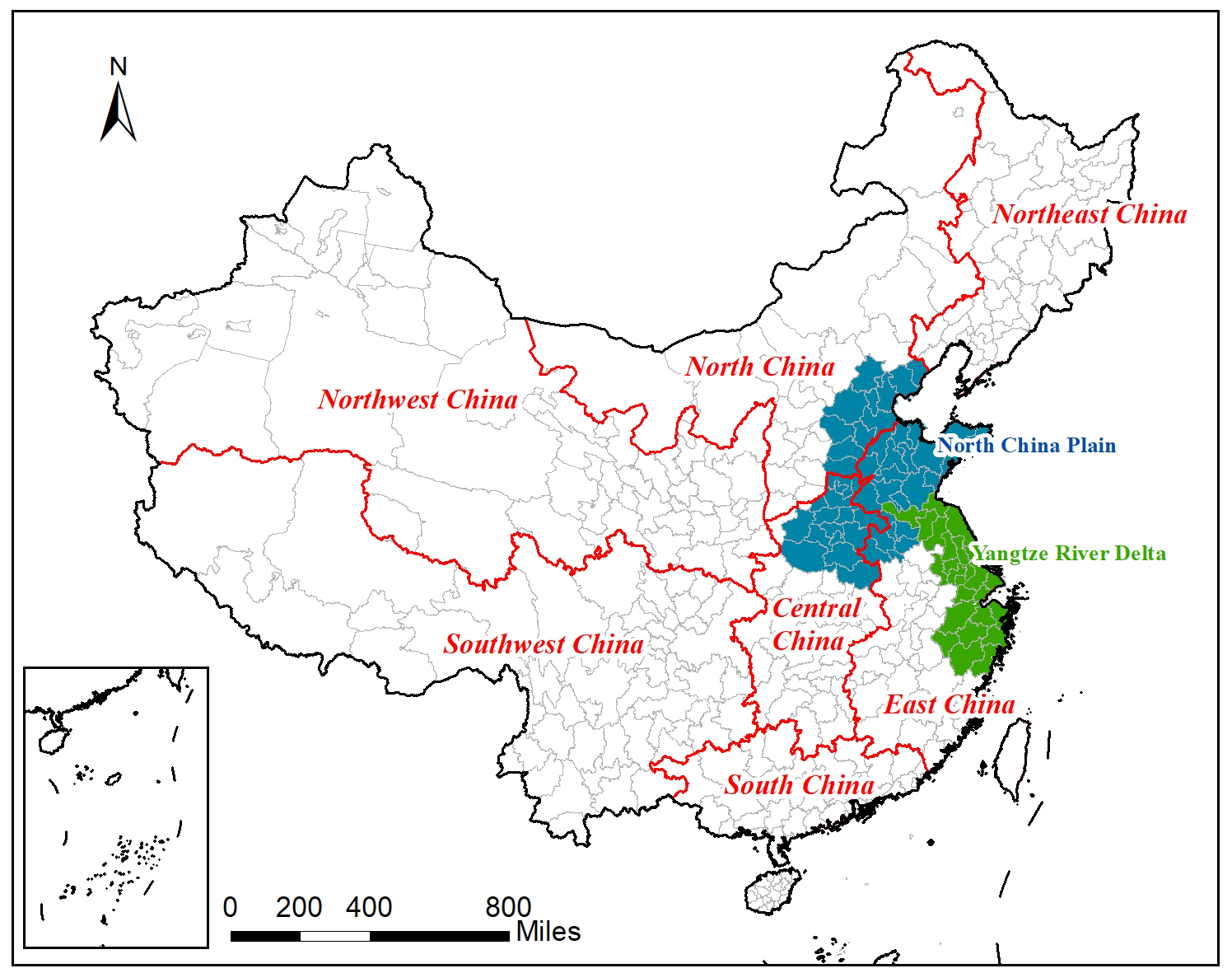

Supplement: Supplementary file 2 [file Image_2.JPEG]
