# Supplementary material for: Spatio-Temporal Heterogeneity of the Relationships Between PM2.5 and Its Determinants: A Case Study of Chinese Cities in Winter of 2020
Source: Front Public Health. 2022 Apr 11;10:810098. doi: 10.3389/fpubh.2022.810098 (PMC9035510; doi:10.3389/fpubh.2022.810098)
Supplement: Supplementary file 3 [file Table_1.DOCX]

**TABLE S1** Relative change rates of 7 driving factors in main haze polluted areas before and after city closure (%).

| Region | Natural factors | | | | Anthropogenic factors | | |
| --- | --- | --- | --- | --- | --- | --- | --- |
|  | sp | rh | tem | ws | ITA | DVI | DVP |
| NCP | 0.06 | -8.22 | 0.40 | 11.32 | -56.60 | -22.20 | -11.80 |
| YRD | 0.06 | -5.43 | 0.29 | 11.33 | -59.92 | -34.00 | -32.94 |
| NEC | 0.05 | -3.71 | 0.12 | 9.56 | -48.34 | -20.41 | 0.90 |
| Xinjiang | 0.05 | -6.93 | 0.36 | 8.97 | -59.87 | -3.60 | 0.14 |

Note: NCP refers to North China Plain. YRD refers to Yangtze River Delta. NEC refers to Northeast China.
